# Statement of retraction

**DOI:** 10.1080/10717544.2022.2088154

**Published:** 2022-07-01

We, the authors, Editors and Publisher of the journal *Drug Delivery*, have retracted the following article:

“Synergism of cisplatin-oleanolic acid co-loaded hybrid nanoparticles on gastric carcinoma cells for enhanced apoptosis and reversed multidrug resistance” 10.1080/10717544.2019.1710622

Since publication, the authors noted that the article contains errors/incomplete information related to the *in vivo* antitumor efficacy of different drug delivery systems presented in the article. Consequently, these errors undermine the conclusions drawn in this paper and request that the paper is retracted.

The authors alerted the problems to the Editor and Publisher, and all have agreed to retract the article to ensure the integrity of the scholarly record. The authors apologize for this error.

We have been informed in our decision-making by our policy on publishing ethics and integrity and the COPE guidelines on retractions.

The retracted article will remain online to maintain the scholarly record, but it will be digitally watermarked on each page as ‘Retracted’.

